# Robust Analysis of Network-Based Real-Time Kinematic for GNSS-Derived Heights

**DOI:** 10.3390/s151027215

**Published:** 2015-10-26

**Authors:** Tae-Suk Bae, Dorota Grejner-Brzezinska, Gerald Mader, Michael Dennis

**Affiliations:** 1 Department of Geoinformation Engineering, Sejong University, Seoul 143-747, Korea; 2 Department of Civil, Environmental and Geodetic Engineering, The Ohio State University, Columbus, OH 43210, USA; E-Mail: grejner-brzezinska.1@osu.edu; 3 National Geodetic Survey Department, Silver Spring, MD 20910, USA; E-Mails: gerald.l.mader@noaa.gov (G.M.); michael.dennis@noaa.gov (M.D.)

**Keywords:** GNSS, real-time kinematic (RTK), network RTK, GNSS-derived heights, GPS benchmarks

## Abstract

New guidelines and procedures for real-time (RT) network-based solutions are required in order to support Global Navigation Satellite System (GNSS) derived heights. Two kinds of experiments were carried out to analyze the performance of the network-based real-time kinematic (RTK) solutions. New test marks were installed in different surrounding environments, and the existing GPS benchmarks were used for analyzing the effect of different factors, such as baseline lengths, antenna types, on the final accuracy and reliability of the height estimation. The RT solutions are categorized into three groups: single-base RTK, multiple-epoch network RTK (mRTN), and single-epoch network RTK (sRTN). The RTK solution can be biased up to 9 mm depending on the surrounding environment, but there was no notable bias for a longer reference base station (about 30 km) In addition, the occupation time for the network RTK was investigated in various cases. There is no explicit bias in the solution for different durations, but smoother results were obtained for longer durations. Further investigation is needed into the effect of changing the occupation time between solutions and into the possibility of using single-epoch solutions in precise determination of heights by GNSS.

## 1. Introduction

Since the development of a centimeter-level accuracy positioning techniques in real-time, based on the integer ambiguity resolution of the Global Navigation Satellite System (GNSS) measurements, there have been great advances in real-time kinematic (RTK) applications. Under the conventional RTK, raw measurements for the reference station are transmitted to the rover for the integer ambiguity resolution and final coordinate estimation [1]. Therefore, most common errors can be cancelled out by differential techniques. However, single-base RTK baseline length should not exceed 20 km in practice [2] due to distance-dependent biases, such as ionospheric and tropospheric delays. New approaches, such as Virtual Reference Stations (VRS), Flächen Korrektur Parameter (FKP), and/or Master-Auxiliary corrections (MAX) were introduced in mid-1990s. These new approaches are known as network RTK (NRTK or RTN), and were successfully used for more than a decade in many applications, especially navigation and surveying.

The aim of RTN is to minimize the influence of the distance-dependent errors from a network of permanent GNSS receivers [3] on estimated coordinates at the rover. Precise models of the error sources derived from the whole reference network are interpolated at the rover’s position [4]. The typical RTN procedure consists of (1) network ambiguity resolution at the master control, based on the known coordinates of the reference stations; (2) estimation of distance-dependent correction model coefficients; and (3) transmission of the coefficients to the user (or rover). Real-time modeling of a reference network and reliable instantaneous ambiguity resolution are required in a short period of time [5,6,7,8]. Due to the different temporal variations of the dispersive and non-dispersive errors, the update rate of the coefficients should be adequately selected (for example, every 10 s for ionospheric delay and 60 s for orbit and tropospheric errors). Several interpolation methods, such as linear interpolation and least-squares collocation (LSC), have been analyzed in detail [3,9]. All methods can significantly reduce the distance-dependent biases in the measurements with a similar performance or comparable accuracy levels. The state-space modeling to better represent the error characteristics (or physical error sources) was suggested by [10], where all error components can be consistently modeled with high accuracy.

The performance analysis, including quality assurance, of RTN was evaluated by many researchers, mostly focusing on the accuracy of the interpolated corrections, positioning accuracy, and initialization time required for precise positioning [11]. Most RTN methods currently used in the industry, such as VRS, FKP, and MAX, provide comparable performance in terms of accuracy and precision of the final solution [3,12], while some differences exist with regards to the processing load, the application of the interpolation algorithm, and the bandwidth of the correction information [13]. It is worthy of notice that the addition of GLONASS observables does not necessarily mean a significant improvement in the RTN results [14]. It is also known that the Radio Technical Commission for Maritime Services (RTCM) version (2 or 3) and/or new proprietary format for the transmission of the corrections have no immediate relationship with the accuracy of the real-time solutions [9,15].

National Geodetic Survey (NGS) guidelines for establishing GNSS-derived ellipsoidal (NGS-58) and orthometric (NGS-59) heights were published in 1997 and 2008, respectively [16,17]. Since then, there have been tremendous advancements in GNSS, particularly in receiver/antenna technology, as well as data processing algorithms and software. In addition, GNSS modernization, including new signals, is underway, and new constellations of GNSS with better satellite orbits are also available. The vertical reference frame, the North American Vertical Datum of 1988 (NAVD 88), was realized based on a nationwide passive network determined using differential geodetic leveling. With recent advances, such as precise hybrid geoids (for example, GEOID12A), the costly, time-consuming leveling process can be replaced by GNSS-derived heights, though the extent depends on the specific application.

Given the recent advancement in real-time technology, new specifications and guidelines are needed for reliable determination of GNSS-derived heights, including consideration of data collection methods and other factors. While most of the studies on the performance of RTK/RTN deal with the accuracy and/or precision of the final coordinates in a controlled environment [2,3,7], we tested the applicability of RTN for height determination using Continuously Operating Reference Station (CORS) networks under realistic field conditions. Since the performance of the RTN is related to many factors, we also focused on the effect of different types of antennas, baseline lengths, solution types, and data collection method of RTN.

## 2. Data Collection

**Figure 1:**
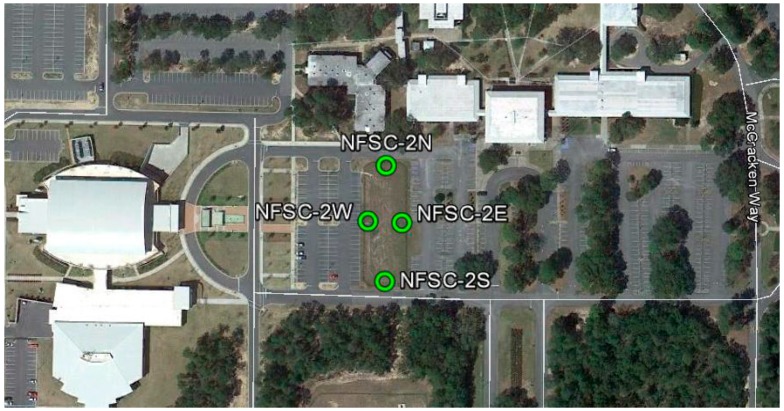
Plan view of new marks in Phase 1 [18].

In this research, two types of experiments were conducted in late 2013 [18]: Vertical Precision phase (Phase 1) and Height Modernization GPS Benchmarks (GPSBMs) phase (Phase 2). During Phase 1, the effect of different receiver/antenna combinations and the repeatability of the solutions were investigated using both static and RT solutions. Four new marks were set up in Niceville, Florida within a diamond shape, separated by about 25 m (from EAST to WEST) and 70 m (from NORTH to SOUTH). As can be seen in Figure 1, these four marks have different multipath conditions, that is, some obstructions in the north and south and a slight possibility of multipath by trees on the east. The final leveled heights are summarized for reference, which denotes the relative heights with respect to the lowest station on the east (Table 1).

**Table 1:** Leveled heights of study site monuments (Phase 1) with respect to the lowest station (NFSC-2E) [18].

Station	Height Differences (m)		Ellipsoid Minus Leveled Ht Diff (m)	Geoid Heights (m)	
	Leveled	Ellipsoid		GEOID12A	USGG2012
NFSC-2E	0.0000	0.0000	0.0000	−27.348	−28.528
NFSC-2N	+0.0032	+0.0041	+0.0009	−27.347	−28.527
NFSC-2S	+0.0180	+0.0199	+0.0019	−27.348	−28.528
NFSC-2W	+0.4387	+0.4407	+0.0020	−27.348	−28.527

The RTN involves multiple reference stations, either the CORS stations or a temporary network established for a specific purpose. The RT data can be categorized into three groups in this study: multiple-epoch single-base RT (RTK), multiple-epoch network RT (mRTN), and single-epoch network RT (sRTN). The RTN solutions were obtained by either GPS only or GNSS (GPS+GLONASS) with different occupation times, but only the GNSS case is considered in this study. For the reference coordinates of the static survey, each site was continuously occupied for 72 h (three days), followed by the antenna swap for the next session (see Table 2). Note that the same receiver/antenna combination is used for all RT solutions of Phase 1. Data collection interval is 1 Hz for both post-processing and real-time (RT) solutions. The mRTN solutions consist of a series of six positions with different durations of five, 30, 60, 180, 300, and 600 s, respectively.

**Table 2:** Receiver and antenna information for static surveying.

	EAST	NORTH	SOUTH	WEST	Note
Set 1	Zephyr Geodetic 2 RoHS	Zephyr-Model 2 RoHS	GNSS-Ti Choke SCIS	R8 GNSS/SPS88x Internal	
	TRM57971.00 NONE	TRM57970.00 NONE	TRM59900.00 SCIS	TRMR8_GNSS3 NONE	IGS model
	R7 GNSS	R7 GNSS	R7 GNSS	R8 Model 3	Receiver
Sets 2–4	Receiver/antenna combination moves in the following order: NORTH ➔ EAST ➔ SOUTH ➔ WEST ➔ NORTH				

**Figure 2:**
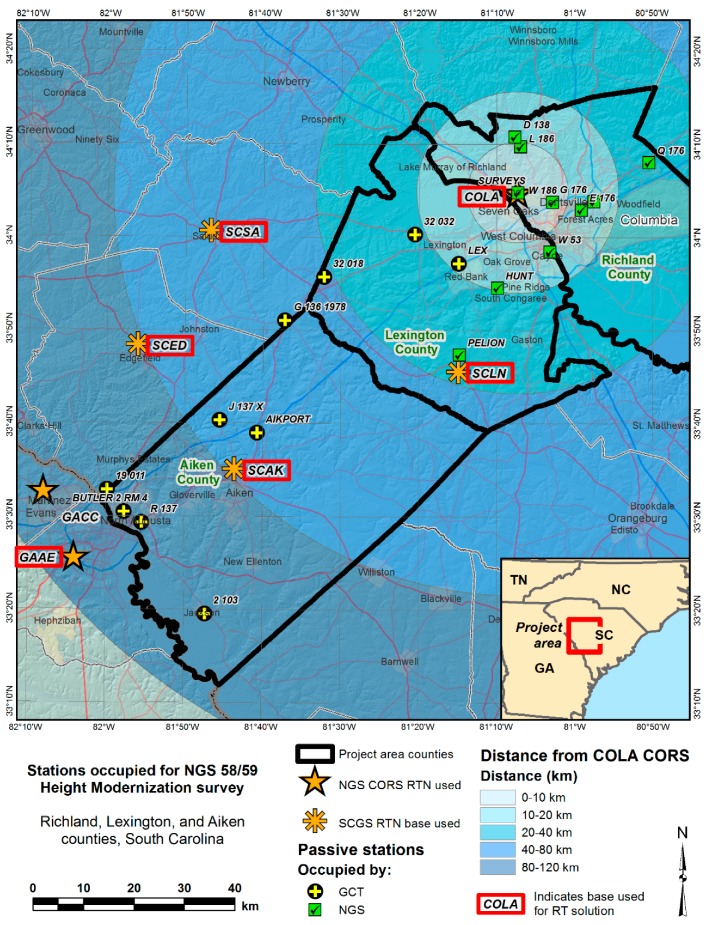
Study area for Phase 2 [18].

In Phase 2, the currently operating CORS network (approximately 35 × 130 km), in the vicinity of Columbia, South Carolina (Figure 2), was used to investigate the effect of the baseline lengths from the same reference station as well as RT types on height information. All GPSBM stations have leveled North American Vertical Datum of 1988 (NAVD 88) orthometric heights stored in the NGS Integrated Data Base (NGSIDB). The data were also collected at a one second interval. A total of 12 permanent bases (seven NGS CORS and five base stations from South Carolina RTN) were used in the surveying (some of stations that were not directly referred to were excluded in Figure 2).

### 2.1 Static Surveying

Four sets of static surveys were performed to validate the effect of antenna type on the final coordinates (Phase 1). In addition, the result of static surveying was used as a reference in the following analysis of RT solutions in Phase 1. All static data processing was performed using NGS Online Positioning User Service (OPUS)-Projects, a set of GPS management and processing tools for projects involving multiple sites and occupations [19]. Users can access the system easily via web for data uploading and (limited) customization of the processing strategy. It uses the PAGES software suite [20] for data processing, and visualization and management aids are also provided. Although all static surveying data were collected at 1 Hz rate in this project, a 30 s resampling was performed for the purpose of this study (and is the only sample rate used by OPUS-Projects). The receiver/antenna combination changed for every session, as summarized in Table 2. The static observations for the new marks, down-sampled at 30 s, were uploaded to and processed by the OPUS-Projects server. The static solutions were used as a basis of comparison reference for the RT solutions. Only one station closest to the project site was selected as a hub station and constrained tightly, while two other CORS stations (about 1000 km away from the hub and located in different directions from it) were also included to decorrelate the tropospheric delay. The tropospheric delay was estimated every two hours in a piecewise linear mode, and the elevation cutoff angle was set to 15°.

### 2.2 RT Surveying

RT solutions were obtained from both Phase 1 and Phase 2. The RT solutions in Phase 1 were performed for newly installed marks to test various factors, such as occupation times, baseline lengths, number of reference stations, number of epochs etc. During the RT session of Phase 1, independent of the static session, the receiver/antenna combinations remained the same to preserve the surveying conditions.

**Figure 3:**
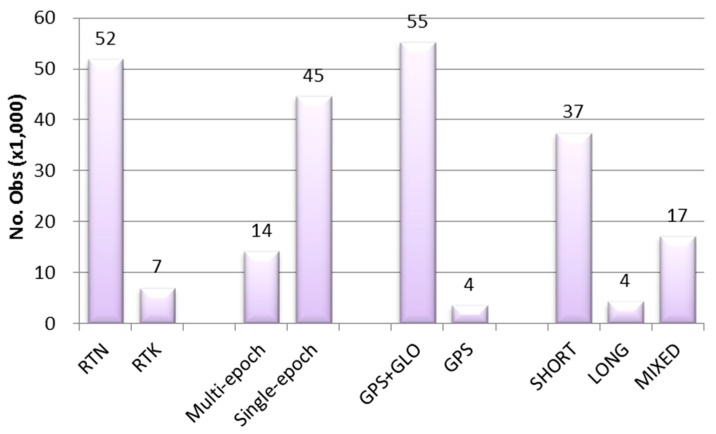
Number of observations by RT types (Phases 1 and 2). SHORT/LONG/MIXED refer to baseline lengths.

On the other hand, in Phase 2, existing GPSBMs with various baseline lengths from seven reference stations were used to verify the relevance of using GLONASS constellation, baseline length, improvement by averaging window, and the effect of the multiple reference stations on the RT solutions. Figure 3 shows the number of observations for each RT type used in this study. RTK solutions were obtained from both Phase 1 and Phase 2. Most of the solutions were based on GNSS, and some GPS-only solutions were obtained in Phase 2. The number of long baseline (~30 km) solutions is very limited, as compared to the count of short baselines (~2 km), and various lengths of baselines were analyzed as well. Unless stated otherwise, all comparisons were accomplished for the ellipsoidal heights in this study.

## 3. Effects of Antenna Types on Static Positioning

**Figure 4:**
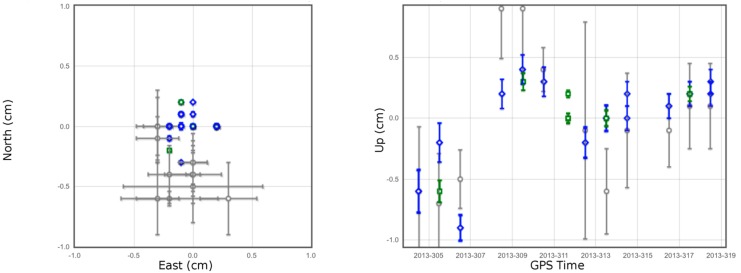
OPUS-Projects processing results for station EAST; horizontal (left); and vertical (right).

Figure 4 shows the horizontal and vertical plots representing the daily solution as an independent site (gray), the daily solution that all sites were processed simultaneously (blue), and the solution for each set as well as the overall estimates (green). While the horizontal components were estimated at a precision (repeatability) of a few millimeters, the vertical one shows a significant variation between sets (up to 1 cm). It should be mentioned here that the estimated final coordinates of each site are based on the most recent reference frame (IGb08) at the mean epoch of data collection (2013.85), and therefore it is necessary to transform the resulting coordinates into NAD83(2011) epoch 2010.00 because all RT solutions in this study were provided in that frame. The transformation between reference frames was accomplished using HTDP (Horizontal Time-Dependent Positioning) by NGS (version 3.2.3).

**Figure 5:**
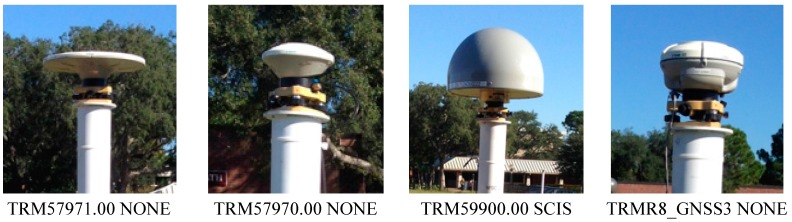
Antennas used in Phase 1 [18].

**Figure 6:**
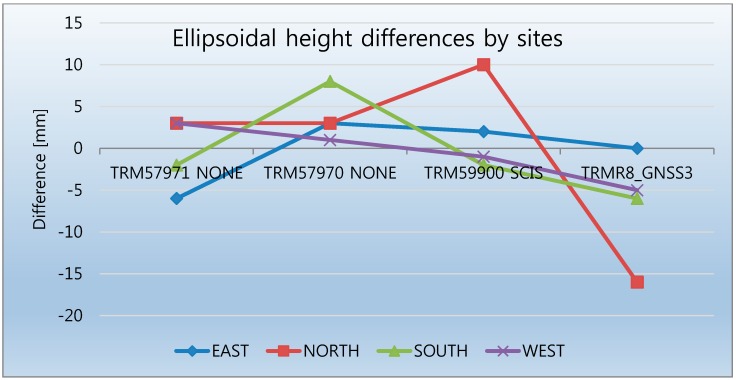
Differences in ellipsoidal heights by site (Phase 1) with respect to the mean OPUS-Projects solution.

Figure 5 shows the antenna types used in Phase 1. The UP component from the same antenna type behaves similarly at two sites, whereas the other two present positive and negative differences, indicating that a similar behavior of the antenna type at each site (positive or negative) cannot be always expected. That is, the TRM57970.00 NONE antenna is positively biased for all sites, and the TRMR8_GNSS3 antenna is offset downward (Figure 6). The difference between the antenna types in the estimated ellipsoidal heights with respect to the mean OPUS-Projects solution reaches up to two centimeters, even though the phase center variation of each antenna type was modeled in the processing software using IGS absolute antenna models.

## 4. Analysis of RT Solutions

### 4.1 Antenna Types

**Figure 7:**
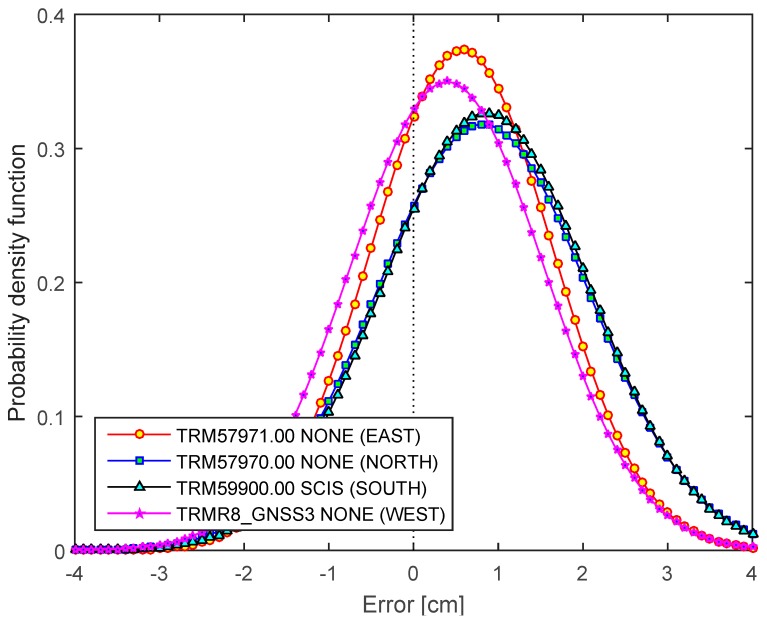
Probability density function (pdf) of RT solutions by antenna types for Phase 1.

Two kinds of experiments were performed in this study in order to verify the effect of antenna calibration on the RT solutions. In Phase 1, mRTN (multiple-epoch network RTK) and sRTN (single-epoch network RTK) solutions were collected for short baselines (~2.1 km) using the same receiver/antenna combinations. The RT solutions were compared with the final solutions determined by the OPUS-Project static surveys (Figure 7). All RT solutions are biased positively (4 to 9 mm), which seems to be related to the error modeling for the tropospheric delay. Two antennas located at station NORTH (smaller ground plane) and SOUTH (Choke ring) provides slightly large biases, which are certainly related to the surrounding environment (signal blocking by trees, see Figure 1). On the other hand, all four antennas perform equivalently under favorable circumstances (no obstructions of reflective objects in close vicinity).

**Figure 8:**
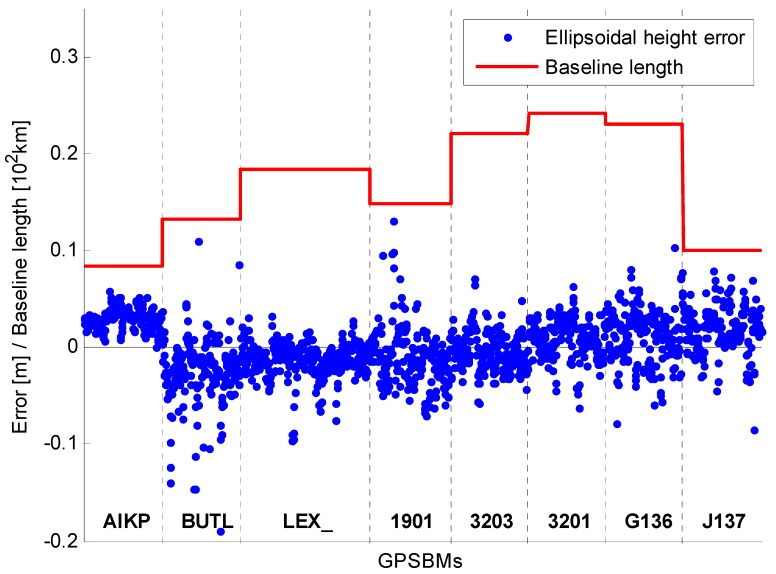
Errors and baseline lengths by antenna type (TRM57971.00 NONE) for Phase 2.

**Figure 9:**
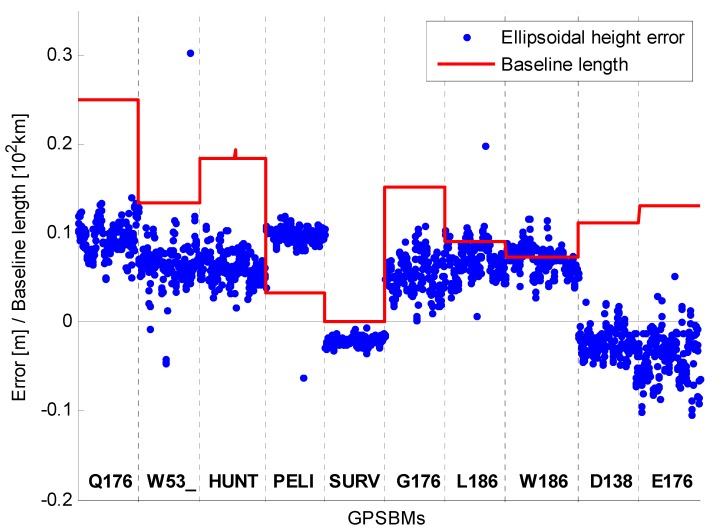
Errors and baseline lengths by antenna type (TRMR8_GNSS NONE) for Phase 2.

It is generally known that the accuracy of the RT solution is inversely dependent on the baseline lengths. The RT solutions from Phase 2 were not analyzed in terms of the baseline lengths because there are 20 different baseline lengths and no significant dependence was observed. In addition, several GPSBMs were obstructed by the surrounding objects (mostly trees), and so it is not easy to separate the effect by baseline lengths from other impacts. Therefore, the RT solutions in Phase 2 were analyzed only by antenna type. Three different types of antennas were used in Phase 2, but one type of antenna was used for two GPSBMs only, therefore, those two GPSBMs were excluded from the analysis. As can be seen in Figure 8 and Figure 9 (mRTN with both GPS+GLONASS and GPS), the differences of the UP component with respect to the mean OPUS-Projects solution were plotted together with the distance to the common base station (COLA). All baselines are less than 25 km, and the reference ellipsoidal heights were obtained from either NGSIDB (NGS Integrated Data Base) or static processing of Trimble Business Center (TBC) software with the vertical recess of the marks taken into account. The built-in antenna showed the most unstable performances, that is, large bias and dispersion were observed even for a relatively favorable surrounding environment.

### 4.2 Baseline Lengths

**Figure 10:**
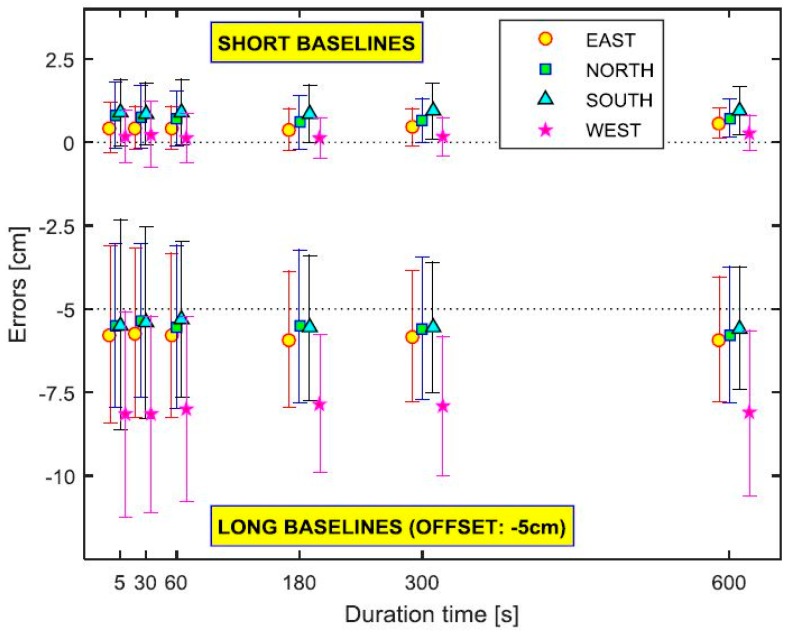
Estimated error bar plot of RTK by duration time and site. The long baselines are intentionally offset by 5 cm for clarity.

For Phase 1, both RTK and RTN solutions were obtained to compare the effect of the baseline lengths. Two temporary bases were established for the reference: FLNV (about 2.1 km away from the project site) and FLFP (~30.6 km). All RT vectors were referenced to the base coordinates determined by the OPUS-Projects using the data collected independently during the RT sessions in order to be consistent. The multiple-epoch RTK solution shows that the relative precision for short baselines (FLNV) as a function of the duration time decreases slowly. The sigma (standard deviation) values given by the RT solutions are generally worse than the calculated precision. The RT solutions from long baselines (FLFP) do not show any particular bias (but with relatively low precision) when compared with the reference solution (Figure 10). The site on the west, however, consistently provides a biased solution for both short (several millimeters) and long baselines (up to 2 cm). Since there are no major obstacles for signal blockage on the west, it is attributed to RT survey procedures for the site. In addition, the statistics of RT solutions from the receiver are overly optimistic for long baselines.

**Figure 11:**
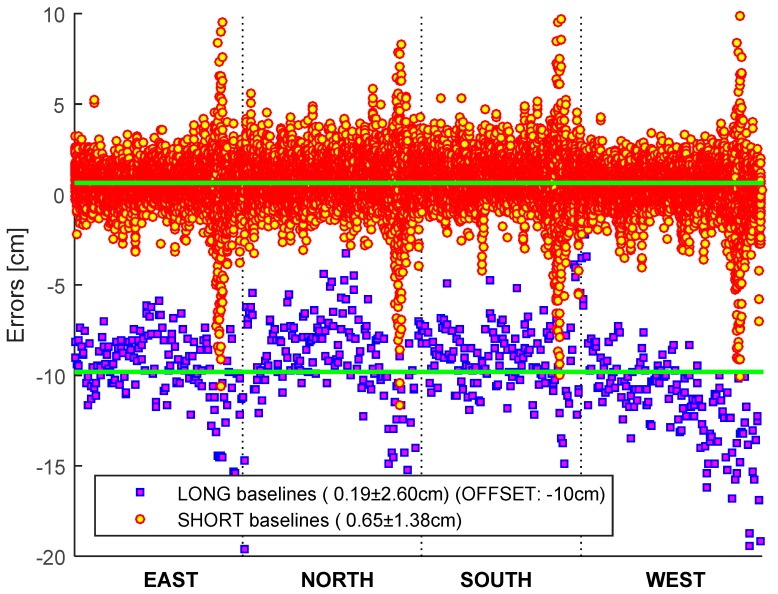
Effect of the baseline length on network RT. The long baselines are intentionally offset by 10 cm for clarity.

Single-epoch network RTK (sRTN) solutions were used to validate the effect of the baseline lengths for RTN in Phase 1. The long baseline solutions were obtained for 2.5 h after almost two and a half days of short baseline solutions, and additional short baseline solutions of 11 h followed (Figure 11). Since the bias for RT solutions can also occur due to a biased reference station height it is not possible to determine whether the bias is from solution or reference station unless the verified reference station coordinates are provided. In addition, special care should be taken not to confuse the antenna reference point with the phase center in the transmitted coordinates of the reference station. It should be noted that there is a significant error in the ellipsoidal height near the end of the session, which is common for all sites. This corresponds to the epoch switching from long baseline to short baseline solutions, which lasts about 30 min. Therefore, the RTN system may not work immediately after switching the reference stations.

### 4.3 Occupation Times

**Figure 12:**
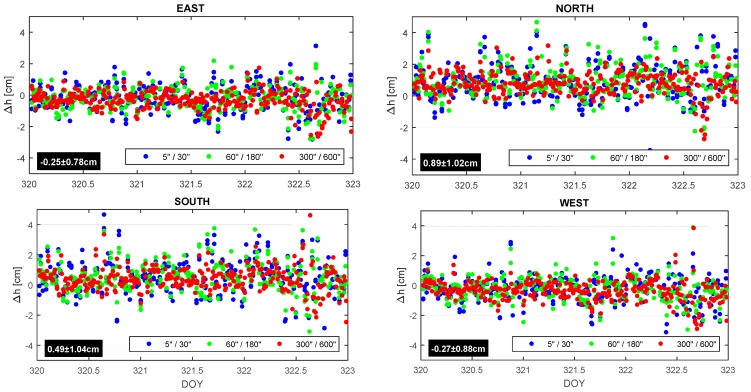
Ellipsoidal height errors plotted for all intervals.

In order to verify the effect of the session duration on RT solutions, the GNSS-based multiple-epoch network RTK (mRTN) solutions were obtained for short baselines (~2 km) in Phase 1. The solutions were alternatively obtained for different occupation of five, 30, 60, 180, 300, and 600 s, respectively. The time series of the ellipsoidal heights for each site is plotted in Figure 12, along with the overall statistics. All observations are grouped according to the session duration and assigned the same color code for two subsequent occupation intervals.

The overall statistics of mean values show a relatively large bias for NORTH and SOUTH due to the surrounding environment, as expected, and their precisions are equivalent. There is no obvious trend in the mean values, except for longer spans, depending on the duration of multiple-epoch solutions, but the variance keeps decreasing as the session duration increases (Table 3).

**Table 3:** Statistics of the multiple-epoch solutions by duration.

**Duration**	5 s	30 s	60 s	180 s	300 s	600 s
**Mean (cm)**	0.27	0.23	0.28	0.25	0.18	0.09
**Std. Dev. (cm)**	1.19	1.15	1.13	1.01	0.94	0.87

## 5. Conclusions

While the advanced technologies in GNSS, including ongoing modernization with new signals, have been introduced into positioning applications, there are no guidelines and specifications for GNSS-derived heights from real-time solutions. Two phases of GNSS RT solutions were intensively analyzed in this study to investigate the possibility of applying network RT solutions for GNSS-derived heights. The RT solutions with four different types of antennas were collected for short baselines in Florida. The performance is generally comparable, but the RT solutions can be biased by up to 9 mm by the surrounding environment. The accuracy of RT solutions is generally dependent on the baseline lengths. However, there seems to be no clear relationship between the baseline lengths and the mRTN solutions in case of GPSBMs, and the built-in antenna is worse than others even for a favorable surrounding environment. For the RTN solution, there is no explicit bias for a baseline up to 30 km (both mRTN and sRTN), and the precision indicator by the receiver is generally overly optimistic, as already pointed out by [2]. The duration of occupations for RTN solutions of up to 600 s shows no obvious difference in the mean values, but the variance of the solutions is inversely proportional to the duration time due to the smoothing effect.

Three different RT solutions of GPSBMs with various baseline lengths were investigated in order to analyze the characteristics of each method. The single-epoch network RTK (sRTN) solution is comparable to the multiple-epoch solutions, which should be investigated further in the future. Possible degradation of quality during transition between different duration times as well as changing the reference station should also be investigated. Since the individually-calibrated antenna phase center variation can deviate up to 1 cm in the up component [21], it may be necessary to consider calibrating the individual antenna for better positioning accuracy of height.

## References

[1] Wanninger L. Introduction to Network RTK. http://www.wasoft.de/e/iagwg451/intro/introduction.html.

[2] Janssen V., Haasdyk J. Assessment of Network RTK performance using CORSnet-NSW. Proceedings of the IGNSS 2011 Symposium.

[3] Berber M., Arslan N. (2013). Network RTK: A Case Study in Florida. Measurement.

[4] Vollath U., Buecherl A., Landau H., Pagels C., Wagner B. Multi-base RTK Positioning Using Virtual Reference Stations. http://www.geosoft.ee/uploads/userfiles/file/ION2000-Paper-MultiBase.pdf.

[5] Li X., Ge M., Douša J., Wickert J. (2013). Real-time precise point positioning regional augmentation for large GPS reference networks. GPS Solut..

[6] Li B., Shen Y., Feng Y., Gao W., Yang L. (2013). GNSS ambiguity resolution with controllable failure rate for long baseline network RTK. J. Geod..

[7] Zou X., Tang W.-M., Ge M.-R., Liu J.-N., Cai H. (2013). New network RTK based on transparent reference selection in absolute positioning mode. J. Surv. Eng..

[8] Zou X., Ge M., Tang W., Shi C., Liu J. (2012). URTK: Undifferenced network RTK positioning. GPS Solut..

[9] Dai L., Han S., Wang J., Rizos C. (2003). Comparison of interpolation algorithms in network-based GPS techniques. Navigation.

[10] Wübbena G., Schmitz M., Bagge A. PPP-RTK: Precise point positioning using state-space representation in RTK networks. http://cors-tr.iku.edu.tr/AutoPlay/geopp_pdf/ion2005_fw_tr.pdf.

[11] Bisnath S., Saeidi A., Wang J.-G., Seepersad G. (2013). Evaluation of Network RTK Performance and Elements of Certification—A Southern Ontario Case Study. Geomatica.

[12] Takac F., Zelzer O. The Relationship between Network RTK Solutions MAC, VRS, PRS, FKP and i-MAX. http://www.nottingham.ac.uk/~iszgwr/PDF%20of%20Rngps10a/05%20GWR/Background/f1.3.pdf.

[13] Janssen V. A comparison of the VRS and MAC Principles for Network RTK. Proceedings of the International Global Navigation Satellite Systems Society.

[14] Martin A., McGovern E. An evaluation of the performance of network RTK GNSS services in Ireland. Proceedings of the International Federation of Surveyors (FIG) Working Week.

[15] Talbot N., Lu G., Allison T., Vollath U. Broadcast network RTK-transmission standards and results. Proceedings of the 15th International Technical Meeting of the Satellite Division of the Institute of Navigation (ION GPS 2002).

[16] Zilkoski D.B., D’Onofrio J.D., Frakes S.J. (1997). NOAA Technical Memorandum NOS NGS-58. Guidelines for Establishing GPS-Derived Ellipsoid Heights (standards: 2 cm and 5 cm).

[17] Zilkoski D.B., Carlson E.E., Smith C.L. (2008). NOAA Technical Memorandum NOS NGS 59. Guidelines for Establishing GPS-Derived Orthometric Heights.

[18] Dennis M. (2014). 2013 NGS 58/59 Update Project: Data Content and Format Summary Report.

[19] NGS opus projects. http://www.ngs.noaa.gov/OPUS-Projects/.

[20] Kass W.G., Dulaney R.L., Griffiths J., Hilla S., Ray J., Rohde J. (2009). Global GPS data analysis at the National Geodetic Survey. J. Geod..

[21] Baire Q., Bruyninx C., Legrand J., Pottiaux E., Aerts W., Defraigne P., Bergeot N., Chevalier J.M. (2013). Influence of different GPS receiver antenna calibration models on geodetic positioning. GPS Solut..

